# Proteomic profiling and identification of significant markers from high-grade osteosarcoma after cryotherapy and irradiation

**DOI:** 10.1038/s41598-019-56024-7

**Published:** 2020-02-07

**Authors:** Rashmi Madda, Chao-Ming Chen, Jir-You Wang, Cheng-Fong Chen, Kuang-Yu Chao, Yu-Min Yang, Hsin-Yi Wu, Wei-Ming Chen, Po-Kuei Wu

**Affiliations:** 1 Department of Orthopedics & Traumatology, Taipei Veterans General Hospital, Taipei, Taiwan; 2 Department of orthopedics, Therapeutical and Musculoskeletal Tumor Research Center, Taipei Veterans General Hospital, Taipei, Taiwan; 3 Orthopedic Department, School of Medicine, National Yang-Ming University, Taipei, Taiwan; 4 Department of Research and Development, National Yang-Ming University, Taipei, Taiwan; 5 Institute of Clinical Medicine, School of Medicine, National Yang-Ming University, Taipei, Taiwan; 6 Instrumentation center, National Taiwan University, Taipei, Taiwan

**Keywords:** Proteome, Proteomic analysis, Bone cancer, Predictive markers, Bone cancer

## Abstract

Biological reconstruction of allografts and recycled autografts have been widely implemented in high-grade osteogenic sarcoma. For treating tumor-bearing autografts, extracorporeal irradiation (ECIR) and liquid nitrogen (LN) freezing techniques are being used worldwide as a gold standard treatment procedure. Both the methods aim to eradicate the tumor cells from the local recurrence and restore the limb function. Therefore, it is essential and crucial to find, and compare the alterations at molecular and physiological levels of the treated and untreated OGS recycled autografts to obtain valuable clinical information for better clinical practice. Thus, we aimed to investigate the significantly expressed altered proteins from ECIR-and cryotherapy/freezing- treated OGS (n = 12) were compared to untreated OGS (n = 12) samples using LC-ESI-MS/MS analysis, and the selected proteins from this protein panel were verified using immunoblot analysis. From our comparative proteomic analysis identified a total of 131 differentially expressed proteins (DEPs) from OGS. Among these, 91 proteins were up-regulated (2.5 to 3.5-folds), and 40 proteins were down-regulated (0.2 to 0.5 folds) (p < 0.01 and 0.05). The functional enrichment analysis revealed that the identified DEPs have belonged to more than 10 different protein categories include cytoskeletal, extracellular matrix, immune, enzyme modulators, and cell signaling molecules. Among these, we have confirmed two potential candidates’ expressions levels such as Fibronectin and Protein S100 A4 using western blot analysis. Our proteomic study revealed that LN-freezing and ECIR treatments are effectively eradicating tumor cells, and reducing the higher expressions of DEPs at molecular levels which may help in restoring the limb functions of OGS autografts effectively. To the best of our knowledge, this is the first proteomic study that compared proteomic profiles among freezing, ECIR treated with untreated OGS in recycled autografts. Moreover, the verified proteins could be used as prognostic or diagnostic markers that reveal valuable scientific information which may open various therapeutic avenues in clinical practice to improve patient outcomes.

## Introduction

High-grade osteogenic sarcomas (OGS) are the most common primary malignant bone sarcomas that distress the bone and forms a matrix and osteoid around the knees^1–3^. It accounts one to three per million each year worldwide and has a high rate of incidence in children and adults^3,4^. Currently, the standard treatment procedures applicable for patients are neoadjuvant chemotherapy drugs combined with surgery, precision diagnostic instruments, and limb salvage operations^5^. At present, there are three reconstructive procedures available after resection of tumors that are affected with major joints, include tumor prosthesis, an osteoarticular allograft, and a composite biological reconstruction. Among these three options, biological reconstruction of allograft and autografts (recycled from the resected autogenic bone segment) technique has been widely implemented and become a gold standard procedure for patients with sarcomas^6^. In order to eliminate the residual tumor cells from recycled autografts extracorporeal irradiation (ECIR) and cryotherapy/liquid nitrogen (LN)freezing are the two commonly used treatment methods employed in the biological reconstruction^7,8^. This technique can improve the regeneration of the bone, help to attain union and subsequent remodeling, and especially it restores limb function by supplying blood, osteogenic cells and proteins to the graft interface.

There is an abundant amount of proteins in the human body play a prominent role in numerous biological and physiological processes. Especially every single protein has a unique function and play a crucial role in organs growth, development, metabolic regulation, disease progression, and pathophysiology. Thus, the altered levels of these proteins are extremely useful in the classification of cells and tissues in disease states^9^. Moreover, Proteomics is a composition of global proteins and their isoforms that helps to understand the different biological mechanisms of cells and organisms^10^. It is an emerging field of science that reveals numerous scientific and pathological information about any clinical specimen’s disease condition and treatment effects. The identified significantly expressed proteins could serve as therapeutic and diagnostic markers for cancers. By using the advanced proteomic technologies, we can identify the differentially expressed proteins (DEPs), and their functions, interactions, and structural changes in any clinical specimen^10^. On top of this, there are no reports available to this date related to the changes in protein expressions after ECIR and cryotherapy/LN-freezing treatments. In order to identify the molecular and proteomic changes after these treatments in recycled autografts of OGS helps to distinguish the status of the disease, and the effect of the treatments. In addition to this, a biomarker plays a significant role in monitoring the disease and provides valuable clinical information regarding the treatment concerning the tumor development, and its progression at the physiological and biological state

There is some evidence demonstrated about the effective irradiation dosage and the levels of protein change among the tumor samples^11^. In addition to this, our recent study has successfully evaluated the preservation of bone morphogenetic protein activity with ECIR and LN-freezing in the tumor-bearing recycled autografts for biological reconstruction^12^. But there is no complete protein profile report on alterations of proteins in recycled autografts especially after treatment with LN-freezing and ECIR. Therefore, we aimed to screen the DEPs (p < 0.05) from treated recycled OGS autografts compared with untreated OGS using high-resolution electron spray ionization liquid chromatography (LC-ESI-MS/MS) and tandem mass spectrometry analysis. The identified DEPs from OGS untreated samples help us to understand the tumor microenvironment, recurrence, metastasis, and prognosis of OGS. On the other hand, the altered protein expressions from OGS treated autografts with cryotherapy/LN-freezing and ECIR will provide crucial information about how both the treatments are effectively playing a key role in treating the autografts by eradicating the tumor cells and restores limb function by preserving its essential proteins. Since recycled autografts are widely being used in the biological reconstruction, this comparative proteomic study will provide numerous clues at molecular, and physiological levels such as bone healing, repair, remodeling, and pathophysiology of OGS tumor. Therefore, the identified protein profiles from our study will open new therapeutic avenues to improve patients’ outcomes in clinical practice.

## Results

**Figure 1:**
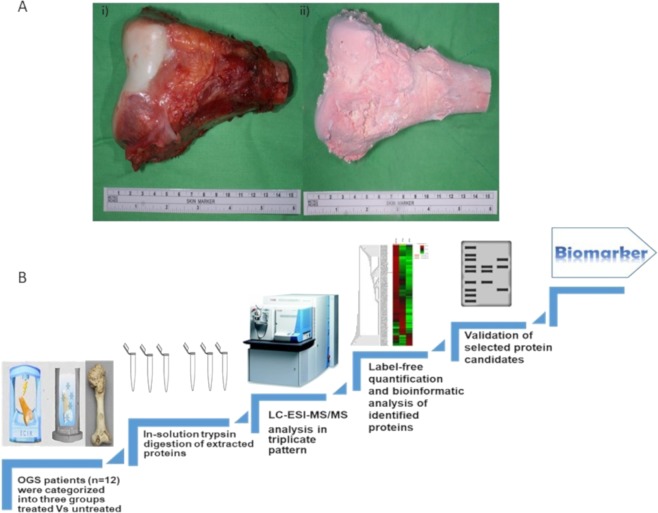
(**A**) Osteosarcoma resected autografts for treatment (i) OGS bone treated using irradiation and (ii) freezing. (**B**) The complete protein profiling workflow of comparative proteomic analysis between treated and untreated samples of osteosarcoma.

Proteins that are expressed significantly after any clinical treatment in human biological fluids have tremendous essential scientific information that helps to reveal potential diagnostic and prognostic features. Biological reconstruction of autografts and allografts is a gold standard procedure using worldwide that helps to fully restores the functions of affected OGS limbs. Cryotherapy/LN-freezing and ECIR are effectively eradicating the tumor cells from OGS bones and the rate of recurrence is very less. Therefore, to elucidate the biological and physiological impact of these methodologies on recycled autografts it is necessary to investigate the proteomic changes to understand the molecular alterations before and after the treatments. Therefore, a total of 36 bone tissue specimens from 12 OGS patients were characterized into three different groups, two groups were subjected as OGS treated such as with cryotherapy/freezing (n = 12) for 15 mins, and the other is ECIR treated at 15,000 gamma irradiations (n = 12), and the third is an untreated group (n = 12) which is a negative control. The complete workflow of our study along with freezing treated and ECIR treated OGS bone illustrations were shown in Fig. 1A,B.

**Figure 2:**
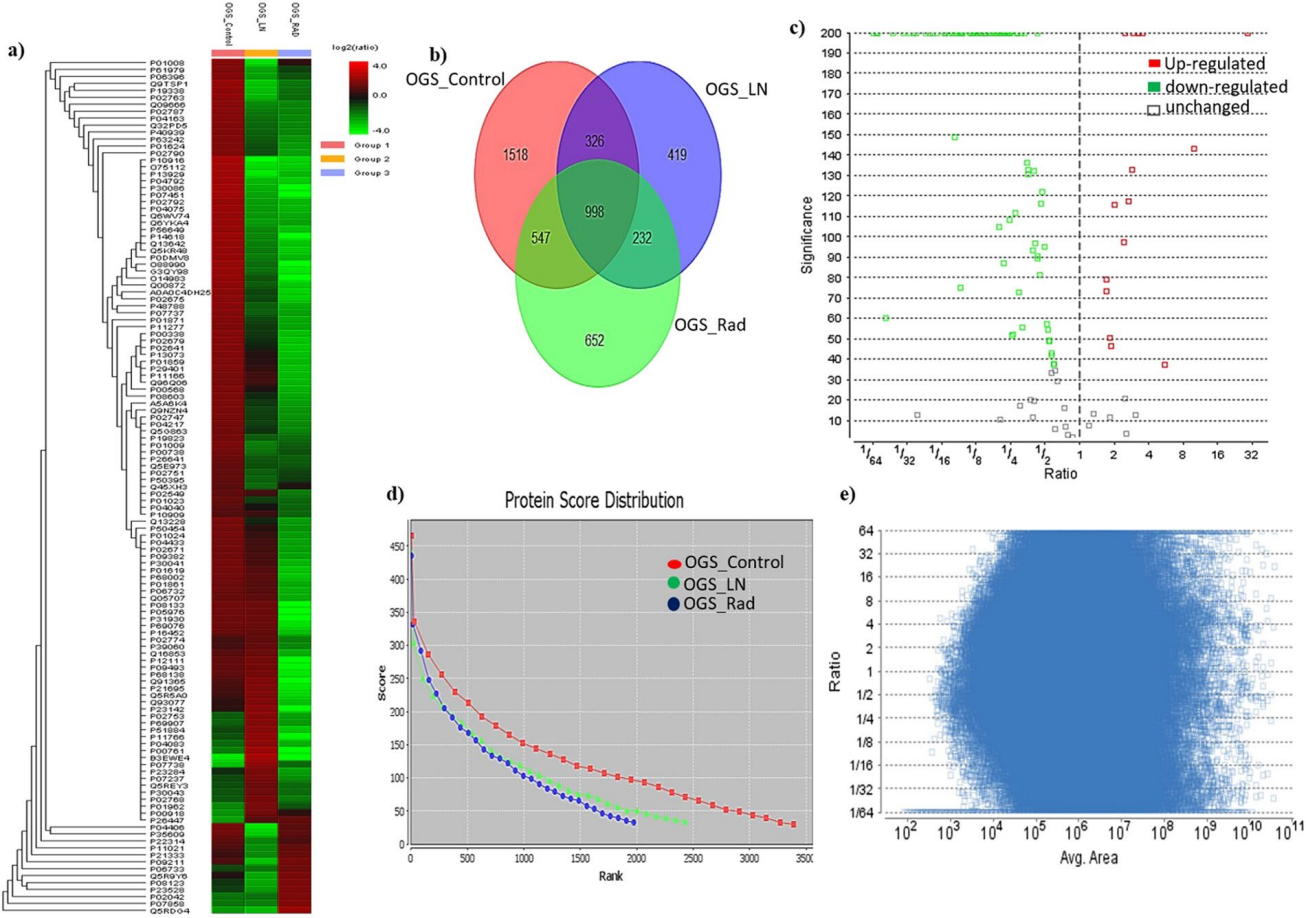
Identification of differentially expressed proteins from high-grade osteosarcoma compared after freezing and irradiation using liquid chromatography and tandem mass spectrometry label-free quantification (**a**) Heat map of OGS comparative proteomic profile among treated and untreated OGS was generated using PEAKS X software. (**b**) Venn diagram showing the differentially expressed proteins from comparative proteomic analysis of OGS control (untreated), and treated with freezing and ECIR. (**c**) Protein volcano plot illustration of significantly identified proteins red dots represents the up-regulated proteins green dots represents the down-regulated and the no colored box represents the unchanged proteins of the comparative analysis. (**d**) The protein score distribution among the three groups. (**e**) The distribution of feature vector ratio by intensity quantified using area under the curve by label-free quantification.

Protein samples were extracted from both treated and untreated OGS groups were subjected to triplicate LC-ESI-MS/MS analysis. Our comparative proteomic evaluations identified a total of 1518 proteins from untreated OGS, and 419 proteins from freezing treated, and 652 proteins from ECIR treated samples. In a heatmap generated by PEAKS Q software version X shows the differences in protein abundances of the identified proteins and their fold changes in Fig. 2a. The commonly identified proteins from three-groups are 998, and the commonly identified proteins among OGS control and freezing treated were 326. On the other hand, between OGS control and ECIR treated equally identified proteins were 527. When compared to OGS freezing and ECIR groups 232 proteins were identified (Fig. 2b). In order to obtain the highly significant differentially expressed proteins from our study, we filtered these proteins using the false discovery rate (FDR) of <0.1%, and the highest protein scores of >70 with a significance score of <20, and at least 2 up to ten unique peptides should be identified (Fig. 2c & Supplementary Table 1). Therefore, from our analysis, we have successfully identified a total of 131 proteins were significantly expressed in OGS treated vs untreated. About 99% of identified proteins from the three groups were quantified and the ratios were measured between triplicate individual samples and the results were highly correlated (r = 0.91, Supplementary Table 2). Among 131 proteins, 90 were up-regulated with >3.5–1.5-fold (p < 0.05 or 0.01) in OGS untreated/treated samples. The complete list of significantly identified up-regulated proteins from OGS control compared to the treatment groups was shown in Table 1. On the other hand, the complete list of 40 proteins which are down-regulated with <0.2–0.5 folds (p < 0.01 or 0.05) were presented in Tables 2 and 3. All the identified proteins and their abundances were compared and quantified using one-way ANOVA and the statistical significances were measured as   0.01 to 0.05.

**Table 1:** List of Up-regulated proteins in OGS untreated/control compared to OGS treated with LN-freezing and ECIR groups.

S.NO	Description	Accession	Avg. Mass	FDR	Coverage (%)	#Peptides	#Unique	OGS Control Area	OGS LN Area	OGS RAD Area	P-Value
**Up-regulated proteins in OGS untreated/control compared to the treatment groups**											
1	Serotransferrin	P02787	77064	<0.02	36	24	2	5.94E + 08	1.86E + 08	1.66E + 08	0.03
2	Collagen alpha-1(XIV) chain	Q05707	193513	<0.01	7	10	6	5.87E + 07	5.72E + 07	1.44E + 07	0.03
3	Spectrin beta chain	P11277	246466	<0.02	1	2	1	8.57E + 06	4.67E + 06	1.78E + 06	0.0008
4	Alpha-actinin-2	P35609	103854	<0.01	4	4	1	6.28E + 06	7.35E + 05	5.40E + 06	0.0003
5	Sarcoplasmic/endoplasmic reticulum calcium ATPase 1	O14983	110252	<0.02	2	2	1	1.08E + 08	4.02E + 07	8.01E + 05	0.00213
6	Fibrinogen beta chain	P02675	55928	<0.01	31	11	6	4.34E + 08	1.99E + 08	4.75E + 07	0.021
7	Alpha-actinin-3	O88990	103043	<0.02	5	5	1	1.64E + 07	4.81E + 06	1.67E + 05	0.00012
8	Creatine kinase M-type	P06732	43101	<0.01	21	7	3	2.07E + 09	1.71E + 09	3.79E + 08	0.00014
9	Fibrinogen alpha chain	P02671	94973	<0.02	10	8	8	4.76E + 08	3.76E + 08	1.11E + 08	0.00052
10	Apolipoprotein A-I	P02647	30778	<0.01	56	14	4	7.88E + 08	2.59E + 08	3.75E + 07	0.000542
11	Annexin A6	P08133	75873	<0.02	4	2	1	3.01E + 07	2.95E + 07	5.11E + 05	0.002
12	Phosphoglycerate kinase 1	P00558	44615	<0.01	35	13	3	4.56E + 07	2.35E + 07	1.05E + 07	0.002
13	Myosin-binding protein C	Q00872	128294	<0.02	3	3	3	3.36E + 07	1.27E + 07	3.03E + 06	0.001
14	Glyceraldehyde-3-phosphate dehydrogenase	P04406	36053	<0.01	43	13	1	6.27E + 06	4.22E + 05	4.96E + 06	0.00000003
15	Beta-enolase	P13929	46987	<0.02	30	9	2	1.28E + 08	7.23E + 06	1.33E + 07	0.005
16	Fructose-bisphosphate aldolase A	P04075	39420	<0.01	44	13	2	2.06E + 08	3.48E + 07	2.37E + 07	0.005
17	Complement factor H	P08603	139096	<0.02	3	4	3	1.03E + 08	5.96E + 07	2.02E + 07	0.00054
18	Heat shock 70 kDa protein 1 A	P0DMV8	70052	<0.01	12	5	1	1.82E + 07	4.85E + 06	2.15E + 06	0.000569
19	Gelsolin	P06396	85697	<0.02	10	5	1	6.40E + 06	1.36E + 06	2.92E + 06	0.000425
20	Tropomyosin beta chain	P07951	32851	<0.01	23	7	1	1.78E + 07	4.81E + 06	1.44E + 06	0.00041
21	Trifunctional enzyme subunit alpha mitochondrial	P40939	83000	<0.02	4	2	2	1.04E + 07	4.38E + 06	3.19E + 06	0.001
22	Fibrinogen gamma chain	P02679	51512	<0.01	35	12	10	3.06E + 08	2.19E + 08	5.18E + 07	0.001
23	L-lactate dehydrogenase A chain	P00338	36689	<0.02	14	5	1	1.43E + 08	7.39E + 07	1.87E + 07	0.001
24	Transketolase	P29401	67878	<0.01	10	6	1	4.78E + 07	2.89E + 07	6.49E + 06	0.001
25	Carbonic anhydrase 3	P07451	29557	<0.02	13	2	1	2.06E + 08	2.71E + 07	6.53E + 05	0.001
26	Dihydropyrimidinase-related protein 2	Q16555	62294	<0.01	11	6	1	2.73E + 06	8.99E + 05	4.43E + 06	0.001
27	Peroxiredoxin-6	P30041	25035	<0.02	22	4	3	1.19E + 08	8.58E + 07	2.08E + 07	0.001
28	Perilipin-4	Q96Q06	134431	<0.01	2	2	2	1.72E + 07	1.18E + 07	2.83E + 06	0.001
29	Four and a half LIM domains protein 1	Q13642	36263	<0.02	13	4	3	1.19E + 08	3.90E + 07	1.31E + 07	0.001
30	Adenylate kinase isoenzyme 1	P00568	21635	<0.01	28	3	3	6.11E + 07	3.58E + 07	1.00E + 07	0.001
31	Immunoglobulin heavy constant gamma 2	P01859	35901	<0.02	22	8	2	3.16E + 08	1.53E + 08	3.00E + 07	0.001
32	Immunoglobulin heavy constant mu	P01871	49440	<0.01	10	4	1	2.97E + 07	1.47E + 07	5.54E + 06	0.001
33	Troponin I	P48788	21339	<0.02	15	3	3	6.11E + 07	2.60E + 07	1.02E + 07	0.001
34	Immunoglobulin heavy constant gamma 4	P01861	35941	<0.01	43	11	3	3.93E + 08	3.31E + 08	8.71E + 07	0.001
35	Nucleolin	P19338	76615	<0.02	1	1	1	2.07E + 07	2.28E + 06	6.46E + 06	0.001
36	Histone H3	P69076	15402	<0.01	14	3	1	2.09E + 07	2.01E + 07	1.69E + 06	0.001
37	Myosin light chain 1/3 skeletal muscle isoform	P05976	21145	<0.02	11	2	1	2.55E + 07	2.48E + 07	7.31E + 05	0.001
38	Ferritin light chain	P02792	20020	<0.01	23	3	3	4.67E + 09	7.61E + 08	5.25E + 08	0.001
39	Histone H4	Q6WV74	11395	<0.02	50	7	1	8.83E + 07	1.65E + 07	1.30E + 07	0.001
40	Heat shock protein beta-1	P04792	22783	<0.01	33	4	2	2.33E + 08	2.29E + 07	1.69E + 07	0.001
41	Cofilin-1	P23528	18502	<0.02	23	2	2	1.58E + 07	6.81E + 06	2.91E + 07	0.001
42	Troponin T fast skeletal muscle	P02641	33034	<0.01	3	1	1	2.29E + 07	1.26E + 07	2.53E + 06	0.001
43	Heterogeneous nuclear ribonucleoprotein K	P61978	50976	<0.02	3	1	1	1.23E + 07	2.52E + 06	6.43E + 06	0.001
44	LIM domain-binding protein 3	O75112	77135	<0.01	1	1	1	9.52E + 06	4.70E + 05	6.32E + 05	0.001
45	Phosphatidylethanolamine-binding protein 1	P30086	21057	<0.02	12	1	1	5.35E + 07	5.78E + 06	6.36E + 05	0.001
46	High mobility group protein B1	P09429	24894	<0.01	7	1	1	8.70E + 06	1.50E + 06	1.46E + 06	0.001
47	Methanethiol oxidase	Q13228	52391	<0.02	3	1	1	1.15E + 07	6.95E + 06	2.75E + 06	0.001
48	Erythrocyte membrane protein band 4.2	P16452	77009	<0.01	2	1	1	1.41E + 07	1.35E + 07	2.15E + 06	0.001
49	40 S ribosomal protein S19	P39019	16060	<0.02	14	2	2	9.14E + 06	3.59E + 06	2.60E + 06	0.001
50	Antithrombin-III	P01008	52602	<0.01	2	1	1	4.62E + 06	3.01E + 05	2.62E + 06	0.001
51	Alpha-1-acid glycoprotein 1	P02763	23512	<0.02	7	1	1	8.83E + 08	1.21E + 08	3.00E + 08	0.001
52	Solute carrier family 2 facilitated glucose transporter member 1	P11166	54084	<0.01	5	2	1	3.45E + 06	2.29E + 06	5.16E + 05	0.001
53	Voltage-dependent anion-selective channel protein 2	P45880	31567	<0.02	6	2	2	1.90E + 07	1.59E + 07	2.64E + 06	0.001
54	Eukaryotic translation initiation factor 5A-1	P63241	16832	<0.01	8	1	1	1.55E + 07	6.71E + 06	3.84E + 06	0.001
55	Protein S100-A10	P60903	11203	<0.02	18	1	1	2.67E + 07	9.67E + 06	7.50E + 06	0.001
56	Cytochrome c oxidase subunit 4 isoform 1	P13073	19577	<0.01	7	1	1	2.20E + 07	1.27E + 07	2.79E + 06	0.001
57	Immunoglobulin kappa variable 3–11	P04433	12575	<0.02	8	1	1	2.36E + 07	1.71E + 07	5.73E + 06	0.01
58	Immunoglobulin kappa variable 3–20	P01619	12557	<0.01	8	1	1	4.11E + 07	3.25E + 07	5.51E + 06	0.001
59	Immunoglobulin kappa variable 3–15	P01624	12496	<0.02	8	1	1	5.88E + 07	2.81E + 07	1.40E + 07	0.001
60	Immunoglobulin kappa variable 3D-20	A0A0C4DH25	12515	<0.01	8	1	1	2.55E + 06	1.15E + 06	2.07E + 05	0.001
61	Neutrophil defensin 3	P59666	10245	<0.02	10	1	1	1.84E + 07	1.16E + 07	6.38E + 06	0.001
62	Alpha-1-antitrypsin	P01009	46737	<0.01	17	9	6	1.57E + 09	6.85E + 08	7.21E + 08	0.001
63	Complement c1 q	P02747	25774	<0.02	5	1	1	5.86E + 06	3.29E + 06	1.62E + 06	0.001
64	Galectin-1	P09382	14716	<0.01	33	6	6	2.20E + 08	1.59E + 08	5.28E + 07	0.001
65	Profilin-1	P07737	15054	<0.02	26	4	1	9.53E + 07	3.81E + 07	1.89E + 07	0.001
66	Ubiquitin-like modifier-activating enzyme 1	P22314	117849	<0.01	2	1	1	8.17E + 06	3.38E + 06	7.22E + 06	0.001
67	Haptoglobin	P00738	45205	<0.02	26	9	3	2.68E + 07	1.15E + 07	1.45E + 07	0.00005
68	Inter-alpha-trypsin inhibitor heavy chain H2	P19823	106463	<0.01	3	2	1	7.48E + 06	3.67E + 06	3.22E + 06	0.00001
69	Hemopexin	P02790	51676	<0.02	8	2	2	3.22E + 07	1.75E + 07	7.03E + 06	0.00008
70	Spectrin alpha chain erythrocytic 1	P02549	280013	<0.01	2	6	5	1.96E + 07	1.10E + 07	6.10E + 06	0.001
71	Serpin H1	P50454	46441	<0.02	8	2	1	4.41E + 06	2.93E + 06	1.30E + 06	0.0041
72	Pyruvate kinase PKM	P14618	57937	<0.01	28	10	1	1.90E + 06	4.08E + 05	3.86E + 04	0.00001
73	Alpha-2-macroglobulin	P01023	163290	<0.02	7	8	2	4.89E + 07	3.27E + 07	2.53E + 07	0.0051
74	Alpha-1B-glycoprotein	P04217	54254	<0.01	9	4	4	5.42E + 07	3.08E + 07	1.68E + 07	0.001
75	Elongation factor 1-gamma	P26641	50119	<0.02	2	1	1	3.94E + 07	2.17E + 07	2.07E + 07	0.001
76	EH domain-containing protein 2	Q9NZN4	61162	<0.01	6	4	2	2.17E + 07	1.18E + 07	5.73E + 06	0.001
77	Complement C3	P01024	187147	<0.02	12	19	19	4.68E + 08	3.75E + 08	1.59E + 08	0.001
78	Rab GDP dissociation inhibitor beta	P50395	50663	<0.01	3	1	1	1.69E + 07	9.20E + 06	1.17E + 07	0.001
79	Hemoglobin subunit epsilon	Q45XH3	16156	<0.02	22	3	1	6.31E + 09	3.45E + 09	4.93E + 09	0.00001
80	Fibronectin	P02751	262622	<0.01	2	2	1	1.58E + 06	9.03E + 05	1.06E + 06	0.001
81	Catalase	P04040	59756	<0.02	14	5	2	4.19E + 06	7.00E + 06	2.45E + 06	0.001
82	Clusterin	P10909	52495	<0.01	6	2	2	2.51E + 06	2.22E + 06	1.49E + 06	0.001
83	60 S ribosomal protein L18	Q07020	21634	<0.02	5	1	1	5.08E + 06	3.15E + 06	3.04E + 06	0.001
84	Transthyretin	P02766	15887	<0.01	9	1	1	1.52E + 07	1.20E + 07	9.30E + 06	0.001
85	Trifunctional enzyme subunit beta	P55084	51294	<0.02	4	2	1	3.46E + 06	2.13E + 06	1.96E + 06	0.001
86	40 S ribosomal protein S25	P62851	13742	<0.01	7	1	1	4.78E + 06	3.05E + 06	3.82E + 06	0.001
87	Band 3 anion transport protein	P02730	101792	<0.02	13	7	6	2.06E + 08	1.21E + 08	1.07E + 08	0.001
88	Eukaryotic translation initiation factor 3 subunit I	Q13347	36502	<0.01	3	1	1	6.44E + 05	1.95E + 05	3.58E + 05	0.001
89	Carbonic anhydrase 1	P00915	28870	<0.02	21	5	4	2.79E + 08	2.12E + 08	2.24E + 08	0.001
90	Peroxiredoxin-2	P32119	21892	<0.01	40	9	2	2.99E + 07	6.00E + 06	1.78E + 07	0.001

LN: Liquid Nitrogen; OGS: Osteosarcoma RAD: Irradiation/ECIR, FDR: False Discovery Rate.

**Table 2:** List of down-regulated proteins in OGS untreated/control compared to OGS treated with LN-freezing and ECIR groups.

S.NO	Description	Accession	Avg. Mass	FDR	Coverage (%)	#Peptides	#Unique	OGS Control Area	OGS LN Area	OGS RAD Area	P-Value
**Down-regulated proteins in OGS untreated/control compared to treated groups**											
1	Collagen alpha-3(VI) chain	P12111	343668	<0.01	2	6	6	2.44E + 08	2.72E + 08	1.28E + 07	0.001
2	Actin alpha skeletal	P68133	42051	<0.01	43	17	1	2.83E + 07	3.35E + 07	2.21E + 06	0.00021
3	Annexin A2	P07355	38604	<0.01	28	8	1	2.33E + 07	3.73E + 07	3.03E + 06	0.00012
4	Tropomyosin alpha-1 chain	P09493	32709	<0.01	23	7	1	3.36E + 07	3.85E + 07	1.76E + 06	0.00025
5	Hemoglobin subunit alpha	P69905	15258	<0.01	70	15	2	8.97E + 08	2.34E + 09	4.89E + 08	0.0025
6	Annexin A1	P04083	38714	<0.01	15	4	1	1.32E + 07	3.86E + 07	5.46E + 06	0.001
7	Carbonic anhydrase 2	P00918	29246	<0.01	5	1	1	2.86E + 06	9.52E + 06	6.45E + 06	0.001
8	Membrane primary amine oxidase	Q16853	84622	<0.01	8	6	1	1.80E + 07	2.08E + 07	4.50E + 06	0.001
9	Glycerol-3-phosphate dehydrogenase	P21695	37568	<0.01	11	4	1	1.70E + 07	2.62E + 07	1.56E + 06	0.001
10	Lumican	P51884	38429	<0.01	26	9	9	3.52E + 08	6.69E + 08	7.48E + 07	0.001
11	Fibulin-1	P23142	77214	<0.01	3	2	1	1.72E + 06	3.25E + 06	5.65E + 04	0.001
12	Immunoglobulin heavy constant gamma 3	P01860	41287	<0.01	29	11	2	4.41E + 06	5.82E + 06	5.66E + 06	0.001
13	40 S ribosomal protein S11	P62280	18431	<0.01	8	1	1	1.90E + 06	2.73E + 06	7.27E + 04	0.001
14	Cytochrome b-c1 complex subunit 1	P31930	52646	<0.01	5	2	1	5.74E + 06	5.72E + 06	2.17E + 05	0.001
15	Histone H2A	Q93077	14105	<0.01	38	5	1	7.21E + 06	1.22E + 07	1.57E + 06	0.001
17	Bisphosphoglycerate mutase	P07738	30005	<0.01	5	1	1	1.95E + 05	1.93E + 06	6.50E + 05	0.001
18	Adipocyte plasma membrane-associated protein	Q9HDC9	46480	<0.01	6	1	1	3.17E + 06	7.86E + 06	2.85E + 06	0.001
19	Protein disulfide-isomerase	P07237	57116	<0.01	4	2	1	2.37E + 06	4.49E + 06	1.30E + 06	0.007
20	Protein S100-A4	P26447	11729	<0.01	9	1	1	4.77E + 05	1.56E + 06	1.69E + 06	0.001
21	Centrosomal protein of 162 kDa (Fragment)	Q91365	143966	<0.01	1	1	1	4.47E + 05	6.77E + 05	1.06E + 04	0.001
22	Retinol-binding protein 4	P02753	23010	<0.01	5	1	1	1.12E + 06	2.71E + 06	5.42E + 05	0.001
23	Serum albumin	P02768	69367	<0.01	80	70	11	7.61E + 09	6.03E + 09	2.39E + 09	0.001
24	Hemoglobin subunit delta	P02042	16055	<0.01	95	14	2	2.42E + 08	2.52E + 08	6.44E + 08	0.001
25	Collagen alpha-1(XVIII) chain	P39060	178187	<0.01	1	1	1	2.23E + 06	2.73E + 06	1.03E + 06	0.001
26	Vitamin D-binding protein	P02774	52918	<0.01	6	3	3	1.48E + 07	1.75E + 07	7.32E + 06	0.0001
27	Peptidyl-prolyl cis-trans isomerase B	P23284	23743	<0.01	8	2	2	6.12E + 06	1.03E + 07	2.41E + 06	0.00001
28	Alcohol dehydrogenase class-3	P11766	39724	<0.01	3	1	1	1.15E + 06	2.91E + 06	1.06E + 05	0.001
29	Thioredoxin-dependent peroxide reductase	P30048	27693	<0.01	4	1	1	3.14E + 06	5.38E + 06	2.52E + 06	0.00051
30	Flavin reductase (NADPH)	P30043	22119	<0.01	11	2	1	2.63E + 06	4.85E + 06	2.26E + 06	0.00001

LN: Liquid Nitrogen; OGS: Osteosarcoma RAD: Irradiation/ECIR, FDR: False Discovery Rate.

**Table 3:** List of down-regulated proteins after the treatment of OGS irradiation compared to the control.

S.NO	Description	Accession	Avg. Mass	Significance	Coverage (%)	#Peptides	#Unique	OGS Control Area	OGSLN Area	OGSRAD Area	P-Value
**Down-regulated proteins after Irradiation treatment**											
1	Endoplasmic reticulum chaperone BiP	P11021	72333	132	10	5	2	1.48E + 07	5.97E + 06	1.69E + 07	0.001
2	Cathepsin B	P07858	37822	132.88	5	1	1	5.58E + 06	5.05E + 06	1.59E + 07	0.001
3	Glutathione S-transferase P	P09211	23356	200	8	1	1	2.05E + 07	3.86E + 06	2.77E + 07	0.001
4	Collagen alpha-2(I) chain	P08123	129314	130.34	2	1	1	1.73E + 06	6.16E + 05	3.11E + 06	0.001
5	Filamin-A	P21333	280737	121.83	3	6	4	1.07E + 07	5.24E + 06	1.29E + 07	0.001
6	Alpha-enolase	P06733	47169	79.24	29	9	2	1.67E + 07	1.20E + 07	2.87E + 07	0.001
7	Transaldolase	P37837	37540	11.26	9	4	2	5.26E + 06	4.11E + 06	9.62E + 06	0.001
8	Immunoglobulin gamma-1 heavy chain	P0DOX5	49329	11.21	27	11	2	5.13E + 07	2.01E + 07	5.31E + 07	0.001
9	Protein disulfide-isomerase A3	P30101	56782	36.97	5	2	1	1.77E + 07	1.06E + 07	9.84E + 07	0.001
10	Alpha-1-acid glycoprotein 2	P19652	23603	12.51	5	1	1	8.34E + 05	6.97E + 05	2.58E + 06	0.001

LN: Liquid Nitrogen; OGS: Osteosarcoma RAD: Irradiation/ECIR, FDR: False Discovery Rate.

### Protein profile of the differentially expressed proteins

**Figure 3:**
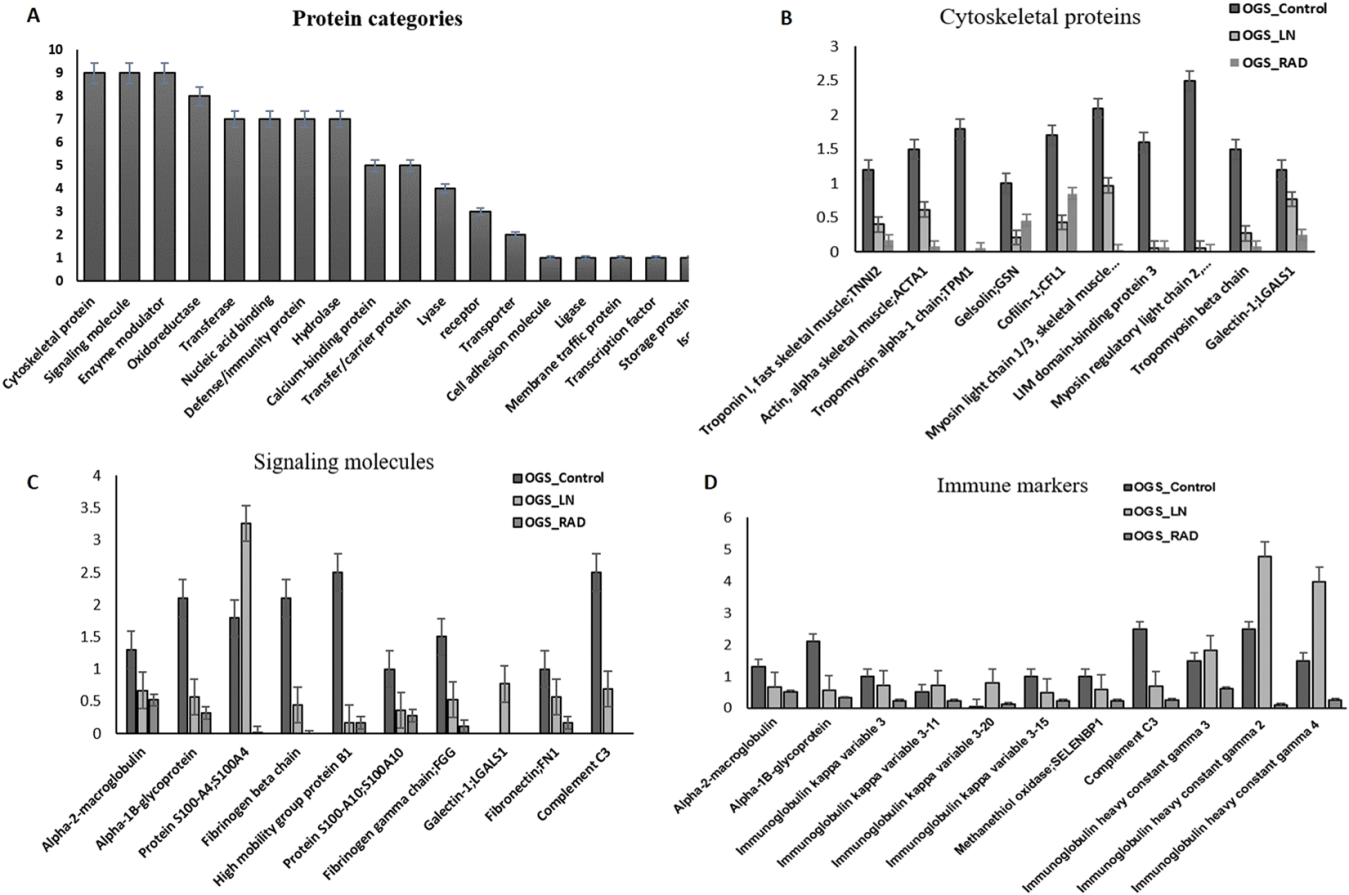
Differential expressions of the identified proteins between treated (OGS n = 12) and untreated (n = 12) D) were categorized based on the functional enrichment analysis. Sample groups statistics (mean ± s.d) obtained for (**A**). Various categories of proteins from this study. (**B**) Altered expressions of cytoskeletal proteins. (**C**) Differential expressions of signaling molecules. (**D**) Immune markers expressions in OGS (all categories were quantified among three groups of OGS samples *p* < 0.01, *p* < 0.05 one-way ANOVA, Mann-Whitney U-test, triplicate analysis).

The functional enrichment analysis categorized the identified significantly altered proteins from this study into more than 10 different classes (Fig. 3A). The top 7 categories which hit the highest number of proteins from our study are cytoskeletal proteins, calcium-binding group, signaling molecules, extracellular matrix proteins (ECM) proteins, immunity or defense proteins, enzyme modulators, transfer, and carrier proteins, etc. We have identified more than 10 cytoskeletal proteins such as Troponin I (TNNI), Alpha-Actin (ACTN1), Tropomyosin alpha-1(TPM1), Gelsolin (GSN) Myosin light and heavy chain (MYL) Cofilin (CFL1), Lumican (LUM), Fibulin-1(FBLN-1), and Galectin (GAL) showed higher expressions levels in OGS control groups. After treatment with LN-freezing/cryotherapy and ECIR treatment, all cytoskeletal protein expressions were reduced from our evaluation except cofilin (Fig. 3B). The expressions of the signaling proteins like Alpha-2-macroglobulin (A2M), Alpha-1B-glycoprotein (A1BG), Protein S100A4, Fibrinogen beta and alpha chains (FGA & FBA) High-mobility group box proteins B1(HMGB1), Galectin (GAL), Fibronectin (FN) and complement C3 proteins from OGS untreated groups showed relatively higher expressions with more than 1-fold (Fig. 3C), and after the treatment with ECIR, and freezing these proteins levels were reduced signifying a numerous clue for further studies.

Over 10 immune/defense proteins (Fig. 3D) have been identified from our analysis, which plays an important role in inflammation, regulation of the immune system and immunotherapy. Most of the proteins associated with the immune system were dysregulated in untreated OGS groups. Surprisingly, after freezing treatment, the heavy and light chain expressions of immunoglobulins were increased. On the other hand, after irradiation treatment, most of the defense proteins were drastically reduced. Additionally, most of the enzyme modulators showed increased expressions with 1.5 to 2.5 folds in OGS control than the treatment group. Other promising results were ECM and calcium-binding proteins which are identified with 2.5 to 3.0 folds elevated in untreated groups and reduced expressions (0.5 to 1.0 folds) were observed in treatment groups. Moreover, from the literature, we came to understand that these proteins play a prominent role in OGS pathogenesis, cell cycle regulation, injury, tissue remodeling, and healing process. This led us to the next confirmation analysis to determine the expression levels of ECM proteins in OGS.

### Gene ontology enrichment analysis

**Figure 4:**
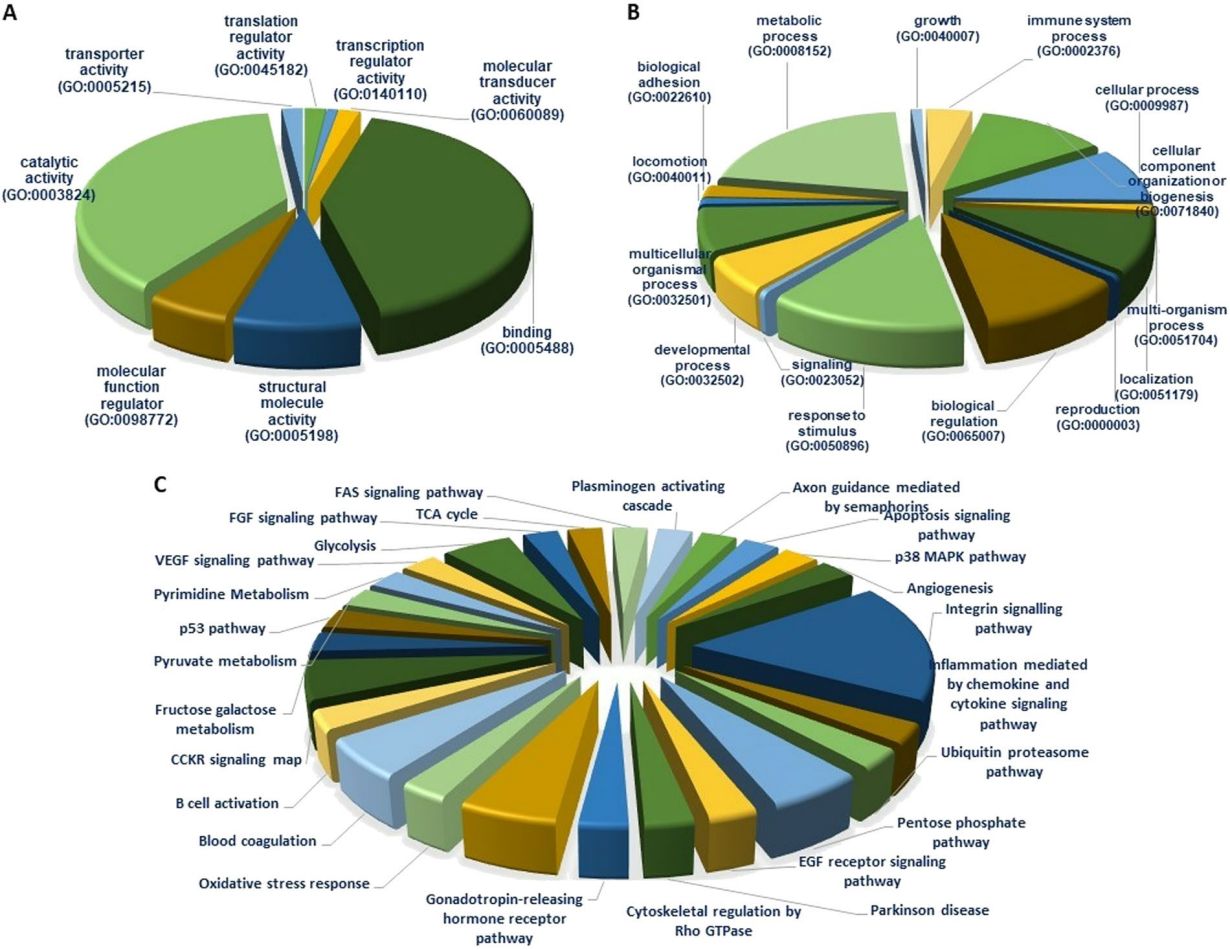
Functional enrichment analysis of identified proteins from comparative proteomic analysis of OGS. using DAVID, KEGG and PANTHER. (**A**) Biological function. (**B**) Molecular functions. (**C**) Identified proteins involved in various important pathways

The functional enrichment analysis of Gene Ontology (GO), Protein Analysis Through Evolutionary Relationships (PANTHER), and Database Annotation Visualization, and Integrated Discovery (DAVID) were performed with the total proteins identified from OGS revealed that the identified proteins were involved in various crucial biological and molecular functions such as biological regulation (22%), metabolic process (36.4%), developmental process (10%), cellular component organization (20.9%) and multicellular organismal process (12.7%). The identified proteins intricate in significant molecular functions such as catalytic activity (38%), binding (41%), molecular function regulation (5.50%), transcription regulation activity (1.8%), etc. The top ten significantly enriched GO biological process and the identified proteins involvement in various molecular functions were shown in Fig. 4A,B.

## Pathway Analysis

The identified DEPs from our study are involved in several significant pathways which play an important role in tumor growth and progression was revealed by Ingenuity Pathway Analysis (IPA) evaluations. Majority of the screened proteins from our study were involved in integrin signaling (17%), inflammation-mediated chemokine and cytokine signaling (3%), and cytoskeletal regulation (8%), etc. Next majority of the significant proteins involved in apoptosis signaling (3%), EGF receptor signaling (3%), VEGF signaling (3%), and p53 pathway (2.5%). Additionally, the greatest number of key proteins and enzyme modulators were taken part in glycolysis pathways (8%) as illustrated in Fig. 4C.

### Protein-protein interaction (PPI) networks

**Figure 5:**
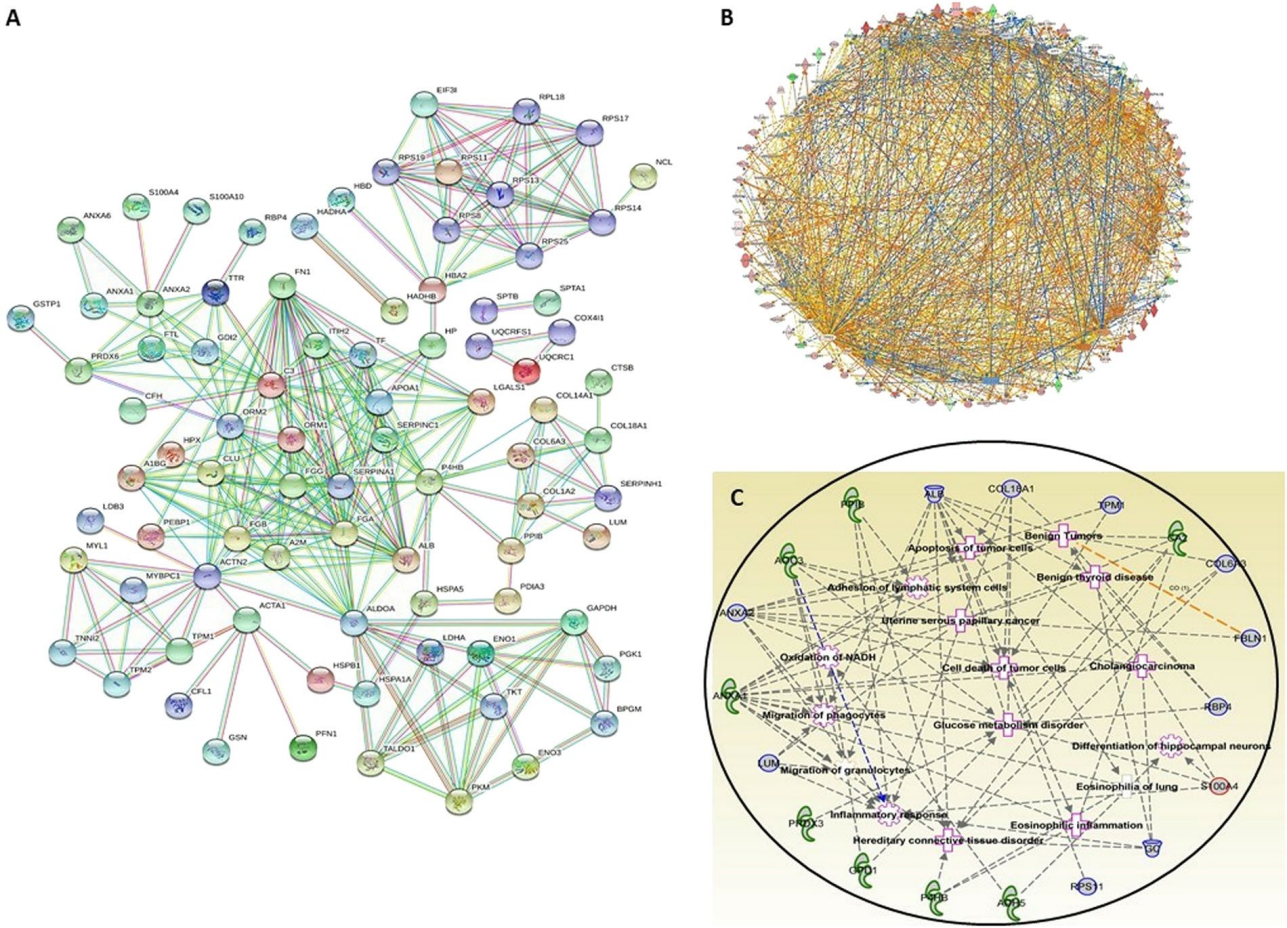
Protein-Protein Interaction (PPI) analysis of the identified proteins from comparative proteomic analysis of OGS using IPA and String networks. (**A**) PPI illustration of tight interaction of altered proteins. (**B**) PPI network showing all the proteins are tightly networked at highest confidence of 0.900 at STRING network analysis.

PPI network analysis was employed by STRING and IPA showed a tight and strong interaction network of all the identified proteins at the highest confidence score of 0.9 was displayed in Fig. 5A,B. Additionally, the IPA analysis also revealed that the identified proteins involved in several carcinomas were demonstrated in Fig. 5C.

### Verification of ECM proteins using western blotting

**Figure 6:**
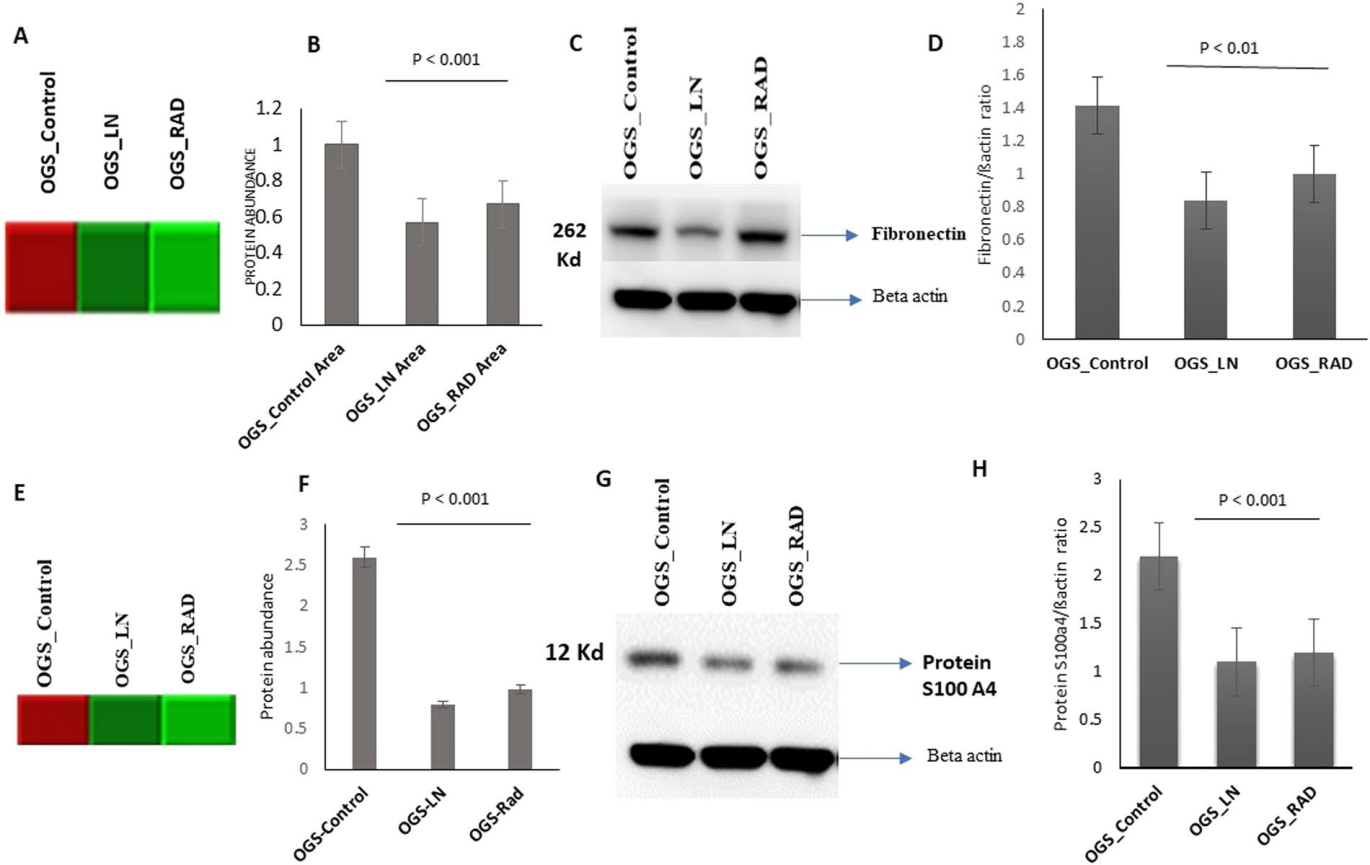
Validation of Fibronectin and Protein S100 A4 expression levels using immunoblot analysis. (**A**) Heat map showing the FN levels evaluated by mass spectrometry. (**B**) Quantification of FN levels from mass spectrometry were showed in a bar chart. (**C**) Immunoblot analysis of validating the FN levels in OGS freezing treated samples compared to OGS untreated and the data was normalized using beta actin. Full length immunoblots were showed in Fig. S1. (**D**) Bar chart showing the validation results of FN. (**E**) Heat map showing Protein S100 A4 levels evaluated by mass spectrometry. (**F**) Quantification of protein S100A4 levels from mass spectrometry were showed in a bar chart. (**G**) Immunoblot analysis validating Protein S100 A4 levels in OGS treated samples, data was normalized using beta actin. Full-length immunoblots were shown in Fig. S2. (**H**) Bar chart showing the quantification values of validation analysis.

ECM proteins significantly contributed to cancer progression (Lu *et al*., 2012). From our proteomic evaluations, ECM’s were significantly down-regulated after freezing and irradiation treatments of OGS. Therefore, from our protein panel, we focused on the expressions of FN and Protein S100A4 which were up-regulated with more than two folds in OGS untreated groups. After the treatment with freezing and irradiation, the expression levels of these proteins were predominantly reduced. Therefore, we have determined to validate and compare the expressions of FN and Protein S100A4 proteins using another set of OGS (n = 6) untreated and treated samples (n = 6) using western blot analysis. Our verification study confirmed the consistent expression patterns FN and Protein S100A4 were significantly correlated with the mass spectrometric analysis (p < 0.01) (Fig. 6A,B).

## Discussion

High-grade OGS is a malignant mesenchymal tumor which produces a matrix or osteoid around the joint either in the distal femur or proximal tibia. It is the most common primary malignant bone sarcoma in adolescents and children^4^. Currently, neoadjuvant chemotherapy drugs in combination with limb salvage surgery is a gold standard treatment option to diagnose OGS. Our recent evidence by Wu *et al*., 2018 concerning biological reconstruction of allografts and autografts have been widely implemented and ECIR and LN-freezing/cryotherapy are being successfully used for treating autografts to eradicate tumor cells from local recurrence^13^. Freezing and irradiation are the most widely used therapeutic modalities to treat autografts^14,15^. Nevertheless, there is a tremendous amount of clinical progress has been made so far in terms of diagnosis, therapeutics and understanding of OGS still there are so many aspects remain unclear, especially concerning the diagnosis, prognosis, tumor development, metastasis, and invasion.

Moreover, the critical challenge in diagnosing OGS is the lack of validated prognostic and diagnostic markers. We hypothesized that freezing and ECIR treatment applications would result in huge changes in the metabolome and transcriptome of bone tissues in the biological reconstruction. In addition to this, there is no evidence available yet regarding the proteomic changes after using both the treatment options on OGS autografts. Therefore, it is necessary to investigate the protein expression changes to understand the molecular and biological levels after using these treatments on OGS. The altered expression levels of proteins will provide numerous keys to open various therapeutic and diagnostic options to treat OGS and improve the current situation efficiently. Based on our vigorous literature search we did not find any proteomics study which has looked at the whole proteome changes on OGS freezing, and irradiation treated samples. Therefore, we aimed to distinguish the DEPs from cryotherapy and ECIR treated autografts compared to untreated samples of OGS (control). From our triplicate proteomic analysis, we have successfully identified a total of 131 proteins with highly significant differences among the three groups. A total of 131 proteins including 90 were up-regulated and 40 were down-regulated with a high score of more than 90 with >2 unique peptides with an FDR of <0.1%. All proteins were consistently identified in triplicate mass spectrometric analysis with the highest accuracy. Based on the literature review, the majority of the up-regulated proteins from our study make a substantial contribution to various crucial pathways which are directly related to the development of tumor growth.

### Cytoskeletal proteins in osteosarcoma

Our proteomic evaluations identified more than 10 altered cytoskeletal proteins showed higher expressions in OGS untreated groups that are down-regulated after freezing and ECIR treatments. TNN1 and TPM1 are actin-binding proteins were elevated in our study are involved in the cytoskeleton’s contractile striated and smooth muscle system^16^. The up-regulated expressions of these proteins are a sign of tumor development and metastasis in OGS. After LN-freezing/cryotherapy, and ECIR treatment these proteins were down-regulated in treated groups. An ezrin family protein MYL that connects major cytoskeletal structures of the plasma membrane showed higher expressions in OGS untreated/control group. Previously, the up-regulated expressions of this protein have been reported to be closely related to Enneking classification and lung metastasis^17^. After ECIR and freezing treatments the higher expressions of MYL were efficiently reduced.

Another actin superfamily ubiquitously expressed member GSN was up-regulated in OGS control. Ma *et al*. group recently reported that the higher GSN expressions are correlated with tumor growth and poor prognosis^18^. Moreover, GSN also supports the growth and metastasis of tumor cells^19^. Interestingly, GSN expression levels were significantly decreased after freezing and irradiation. From earlier evidence reduced expressions of GSN found to reduce human breast, gastric, non-small cell lung cancers^20–23^. Likewise, lower levels of GSN inhibited cell growth, invasion, cell cycle arrest and also played a potential role in OGS as an oncogene^22^. GN is a multifunctional beta-galactoside-binding protein that has been playing a crucial role in carcinogenesis^24^, and has shown up-regulated expressions in untreated groups were showed contrary results in the treated category. Recently, Zhou *et al*. reported that over expressions of GN correlated with Enneking stage of cancer and metastasis occurrence in OGS^25^. Furthermore, over expressions of fibulin have been observed in untreated OGS groups, which is positively correlated with the development of OGS, and its progression, and poor prognosis^26^. Interestingly, after freezing and irradiation fibulin levels have been reduced.

A small proteoglycan-rich in leucine LUM is elevated in OGS untreated groups. It has been demonstrated to contribute to numerous biological and physiological processes. Interestingly the levels have been reduced after the treatment with freezing and irradiation. LUM expressions have been suggested to be positively correlated with the differentiation and negatively associated with OGS progression^27^. Additionally, over expressions of Profilin and Cofilin were also identified in OGS control which can inhibit actin the polymerization^28,29^. It plays a key role in the dynamic change in actin filaments structure and is associated with proliferation, invasion, and metastasis of tumor cells. Our proteomic analysis suggests that both the treatments act effectively on the cytoskeletal proteome level to suppress the higher protein expressions in OGS.

### Signaling proteins expression in OGS

Signaling proteins play a key role in the diagnosis and prognosis of cancer. In our OGS comparative proteomic study, we identified more than 10 significant molecular proteins. All of them showed differential expressions between samples of untreated and treated. Most importantly the calcium-binding Protein S100 A4 and A10 showed higher levels of expression in OGS control were reported earlier in patients with metastasis and poor prognosis^30,31^. Interestingly, after freezing and irradiation treatment, both the proteins were reduced. An ECM family HMGB1 that functions as a signaling molecule and take part in inflammation and carcinogenic ability^32^ has been identified with relatively higher expressions in OGS control compared to the treatment groups. Previous studies reported a poor recurrence and free survival rate associated with the higher expressions in OGS. Another recent study also identified similar results with us demonstrated that the higher expressions of HMGB1 related to cancer development, tumor progression, and metastasis of lymph nodes^33^. On the other hand, we observed reduced levels of HMGB1 in treatment groups revealing that both the treatments successfully reduced the over expressions.

### Immune markers

In response to any disease, the immune system, and its regulation play an outstanding role, especially for malignant tumors, it is a major determinant for the ultimate prognosis. It is evident that the huge cell diversity of the immune niche regulates the OGS microenvironment^34,35^. It has been stated recently that altered IGg’s expressions could promote tumor cell growth^36^. From our analysis, we have identified differential expressions of several IGg’s such as Immunoglobulin kappa variable 3 chains from 3–11, 3–15, 3–20 and Immunoglobulin constant gamma 2, 3 and 4 were significantly down-regulated in OGS control groups were remarkably showed increased expressions after LN-freezing/cryotherapy treatment. A recent study by Kato *et al*. in-kidney cancer patients demonstrated that cryoablation could induce strong immune reactions in tumors with an oligoclonal expansion of antitumor T cells, which circulate systemically^37^. Earlier investigations on cryoablation supports our results that freezing can improve and regulate the immune system process and antitumor response to fight against cancer. By contrast, ECIR treatment drastically reduced IGg’s expressions. Besides, we also evaluated higher expressions of complement C3 and complement factor H in OGS untreated samples, which were reduced remarkably in treatment groups. Since complement is not only an effector of innate immunity but also a contributor to inflammation, hemostasis, adaptive immune response and regulation of the immune system process^38^. Recent findings demonstrated that activation of complement has traditionally been considered as a part of the body’s immunosurveillance against cancer^39^. In addition, elevated expressions of A2M, A1BG, and Methanethiol oxidase have also been identified in OGS control samples which were reduced after treatment indicating that freezing can be an effective immunotherapy target for treating OGS.

### Catalytic proteins

A catalytic member Protein disulfide isomerase-1 (PD1) identified with higher expressions in untreated samples of OGS. Based on the literature, PD1 has been abruptly identified in many tissues and expressed during endoplasmic reticulum stress. Xu *et al*. team demonstrated higher levels of PD1 associated with numerous types of cancer cells including kidney, lungs, brain, ovarian and prostate cancers^40^. In our study, PD1 showed reduced after freezing and irradiation treatment suggesting the fact that both treatments are efficiently reducing the elevated levels of proteins. Recent evidence described that lower levels of PD1 expression could increase the survival rate in breast cancer patients^41^. In addition to this, we have also identified several transcriptions and translation factors such as elongation factor gamma, eukaryotic translation initiation factor 3 subunit, carbonic anhydrase 1, band 3 anion translation initiation factor 3 subunit 1, trifunctional enzyme subunit beta, carbonic anhydrase, cathepsin B, EH domain-containing protein 2, Catalase, Cytochrome c oxidase subunit 4 isoform 1 and so on were differentially expressed in our proteomic study. Furthermore, we have also screened some key glycolysis-related proteins such as glyceraldehyde 2-phosphate dehydrogenase, fructose-bisphosphate aldolase, phosphoglycerate kinase, transketolase, and L-lactate dehydrogenase were up-regulated in untreated groups of OGS were reduced their expressions after freezing and irradiation treatments. Therefore, LN-freezing/cryotherapy and ECIR competently reducing the over expressions of critical proteins and preserve them for biological autografting.

### Extra cellular matrix (ECM) proteins

The ECM proteins play a serious role in the development of cancer by regulating the dynamic behaviors of endothelial cells through different receptors of cell adhesion in cytoskeletal organization, remodeling, and tumor angiogenesis^42^. Thus, ECM proteins are promising therapeutic targets for tumors. From our OGS proteomic study, we have focused on a glycoprotein FN from ECM family which showed up-regulated expressions in OGS control samples and reduced after freezing and irradiation treatments. FN has played a prominent role in cell adhesion, differentiation, cell-matrix regulation, and tumor development^43,44^. We, therefore, choose this multifunctional protein for our validation studies and successfully confirmed its levels of expression using immunoblot analysis. FN levels were tremendously reduced after freezing and ECIR, and in fact, freezing showed more prominent results than ECIR. Thus, FN could be a valuable marker for the prognosis and diagnosis of OGS treatment. We have also validated Protein S100 A4, another important protein of interest from our study. This calcium-binding protein promotes metastasis and has been associated with patient’s outcome in various tumor types. Earlier studies reported the overexpression of Protein S100A4 is associated with tumorigenesis, poor prognosis, prediction of metastasis potency and has been stated as a prognostic marker for OGS^45,46^. From our investigations, we observed a significant amount of reduction in protein S100A4 levels after treated with freezing and irradiation. Besides, it can be a valuable marker to predict metastasis, tumor prognosis and development.

In summary, our comparative proteomic study successfully identified more than 10 different protein categories that are significantly altered expressions were identified after freezing and irradiation treatments compared to untreated OGS group. From our evaluations, both the treatments have effectively reduced the expression levels of highly regulating proteins that directly related to tumor development, recurrence, and metastasis. Besides the majority of the identified proteins from our study are associated with various biological, physiological and molecular functions of OGS. Especially most of the protein expressions have been reduced to the required levels in freezing treatment. On the other hand, in the ECIR treatment, some category of proteins such as immune markers, signaling molecules, calcium-binding protein expressions have been drastically reduced (−0.03 to −0.004) or diminished. Exposure to irradiation may cause cell damage that leads to protein degradation could be one of the reasons behind extreme changes in protein expressions with ECIR treatment. At the same time, LN-freezing showed better results than irradiation. However, we would like to emphasize that both the treatment options are successfully reducing overly expressing proteins which helps for the proper functioning of recycled autografts in the biological reconstruction. Abnormal protein levels can increase tumor growth and metastasis. Therefore, to gain longevity and proper function of the biological autografts, it is important to attain the normal levels of important proteins.

## Conclusion

From our comparative proteomic study among OGS treated with cryotherapy/LN-freezing, ECIR and untreated were successfully identified a set of potential protein markers and their tremendous changes. The identified significantly expressed proteins from OGS non-treated groups play a crucial role in the tumor development, recurrence, metastasis and bone matrix formation which provides numerous clues for diagnosis and OGS management. On the other hand, DEPs from treated OGS groups play an important role in various crucial pathways which are directly related to tumor progression, metastasis, and OGS pathophysiology. We believe this is the first work that shows altered expressions of important protein profiles after freezing and ECIR treatment in recycled autografts. This study sheds new light on the role of freezing and ECIR treatments in biological reconstruction. Most importantly, the identified proteomic patterns and the verified protein candidates from our study help us to understand osteosarcoma in biological, physiological and molecular levels that could open various diagnostic avenues in therapeutics.

## Materials and Methods

### Patients and clinical information

This study included a total of 36 high-grade OGS bone tissue samples from 12 patients (male/female; 10/2; age ranging from 23–65 years) were collected from Taipei Veterans General Hospital (VGH-TPE). The collected OGS samples were categorized into three different groups such as LN-freezing, ECIR treated, and untreated OGS groups for comparative proteomic analysis. The tumor bone samples were collected from all the patients during the surgery. The demographic and clinical features of the obtained samples were shown in Table 4. All samples were freshly collected from the operation theatre after the surgery before chemotherapy, radiation and immunosuppressive medication or any treatment. The collected samples were stored at −80 °C for further analysis. Diagnostic criteria of all the collected OGS patient samples was confirmed by a certified surgeon as well as a pathologist by the tissue biopsy examinations. This study and all the materials and methodology was approved by the institutional review board^47^ of VGH-TPE, Taiwan (**IRB Approval No.2019-02-021 A**), and informed consent was obtained from all the patients. This study confirmed and conducted all the experiments according to the guidelines and regulations of IRB.

**Table 4:** Demographic and tumor characteristics of OGS Patients.

Patient number	Gender	Age	OGS Tumor location	Tumor Length	Status
1	M	49	Distal tibia	13.7	AWD
2	M	45	Distal femur	4.3	NED
3	M	23	Distal tibia	9.3	AWD
4	M	36	Proximal tibia	6.2	AWD
5	M	53	Distal femur	8.5	AWD
6	M	55	Proximal femur	13.7	AWD
7	M	65	Distal tibia	15	AWD
8	F	45	Proximal tibia	4.1	NED
9	F	24	Distal femur	9.1	NED
10	M	36	Proximal tibia	9.8	NED
11	M	56	Proximal femur	14.4, 3.1	AWD
12	M	65	Distal tibia	13.3	NED

AWD: Alive with disease NED: No evidence of disease.

### Protein extraction from OGS samples

#### Cryotherapy treated samples preparation

A total of 12 OGS patients bone tissues were collected, and subjected to Cryotherapy/LN-freezing treatment for 15 minutes under complete sterilization conditions^14,48^. All the freeze tissues were kept at room temperature and thawed for 20–25 mins before protein extraction. To extract the total protein all the samples were subjected to pulverized with a mortar and pestle using liquid nitrogen^49^. Then, the samples were transferred to new Eppendorf tubes and kept on ice until ready for extraction. Later, RIPA lysis buffer (50 mM Tris-HCl pH7.2, 150 Mm NaCl,1% NP40, 0.1% SDS, 0.5% DOC, 1 mM PMSF, 25 mM MgCl2) (sigma; R0278) supplemented with a phosphatase inhibitor cocktail (Thermo; 78420) was used to extract the protein from the samples and centrifuged at 13000 g for 15 min. Then, separated the supernatant to new tubes, and the extracted purified protein from all the freezing treated OGS were subjected to total protein concentration determination assays such as BCA and Bradford (Bio-Rad Laboratories, Hercules, CA)^50^.

#### Extra corporeal irradiation (ECIR) treated samples preparation

A total of 12 OGS samples were subjected to ECIR treatment at 15,000 gamma irradiations^51^. After irradiation the samples were subjected to protein extraction using RIPA lysis buffer (50 mM Tris-HCl pH7.2, 150 Mm NaCl,1% NP40, 0.1% SDS, 0.5% DOC, 1 mM PMSF, 25 mM MgCl2 (sigma; R0278)) supplemented with a phosphatase inhibitor cocktail (Thermo; 78420). Later, all the ECIR treated samples were centrifuged at 13000 g for 15 min. Then, separated the supernatant to a new tube and the extracted purified protein concentration was determined using BCA and Bradford assay (Bio-Rad Laboratories, Hercules, CA)^50,52^. On the other hand, the untreated OGS samples protein was extracted by using the same methodology as above without any prior treatment was employed and protein concentration was determined.

#### Protein precipitation and in-solution digestion

We tried to analyze the autogenous OGS host bone grafts for proteomic analysis using (LC ESI-MS/MS analysis using the same methodology as our previous studies^54^. The extracted protein samples from treated and untreated OGS autografts were precipitated with a fourfold volume of 100% ice-cold acetone and incubated overnight at −20 °C. The precipitated samples were centrifuged at 14000 X g for 10 min, and the pellets were dissolved in 100 µl of 25 mM NH4HCO3 with 6.5 M urea (0. 1–1 µg/µl) followed by an in-solution digestion procedure illustrated by earlier groups^53^. All the protein samples were reduced at 37 °C by 100 mM DTT (Dithiothreitol)^53^ for 30–40 min, later alkylated with 200 mM IAA (Iodoacetamide) in the dark at room temperature for 25–35 min, respectively. The proteins were digested overnight (16–18 hours) with sequencing grade trypsin (Promega, Madison, WI, USA; V5111) in 50:1 ratio at 37 °C. The reaction was quenched by adding 2 µl of 50% formic acid (FA) mixed briefly and incubated for 10 minutes. The digest was briefly vortexed and centrifuged then the supernatant containing peptides were collected. The final solution was lyophilized and desalted using C18 zip-tip procedure^54^.

### Nano UPLC and mass spectrometry conditions

A slightly modified mass spectrometry conditions from our previously described method by Madda R *et al*.^55^ were employed successfully in this proteomic study. At 10000 full-width half maximum (FWHM) resolution an interface of ESI-Q-TOF MS/MS was reached as we performed in our earlier studies^55^. An external standard of lock mass BSA was constantly infused using the Nano-ACQUITY auxiliary pump at an interval of 20 secs (lock spray frequency) for calibrating the instrument at a flow rate of 0.25 µl/min. To obtain the accuracy precursor mass error was chosen as <2 ppm and the lock mass data were averaged. By using the positive V mode all the attained peptide spectra were eluted with a scan mass range of 50–200 m/z at a scan time of 1 sec. The digested 400 ng peptides were reconstituted in 3% ACN (Acetonitrile) and 0.1% FA (Formic Acid), then injected into an online nano-ACQUITY, UPLC coupled Q-TOF, Synapt-HDMS mass spectrometer (Waters Corporation, Milford, MA, USA). Next, the peptides were separated using a C18 reverse-phase column (1.7 µm × 75 µm × 250 mm) (Waters Corporation, Milford, MA, USA). A binary solvent system consisted of 99.9% water and 0.1% FA (mobile phase A) and 99.9% ACN and 0.1% FA (mobile phase B). The peptides were initially pre-concentrated and desalted online at a flow rate of 5 µl/min using a 5 µm symmetry C18 trapping column (internal diameter 180 mm, length 20 mm) (Waters Corporation, Milford, MA, USA) with 0.1% FA. After each injection, the peptides were eluted into the Nano-LockSpray ion source at a flow rate of 300 n/L and a gradient of 2% to 40% for 120 min. Later, the column was washed and equilibrated. The digested OGS treated with freezing, ECIR and untreated/control samples were run in triplicates and the data were analyzed by ProteinLynx Global Server 4.2 software (PLGS: Waters Corporation, Milford, MA, USA)^56^. Each sample was injected three times to obtain technical triplicates.

### Protein quantification

We tried to analyze the autogenous OGS host bone grafts for proteomic analysis using high- resolution electron spray ionization liquid chromatography and tandem mass spectrometry (LC-ESI-MS/MS) analysis. The identified proteins from the LC-ESI-MS/MS analysis were quantified using label-free quantification by PEAKS Studio X (Bioinformatics Solutions Inc. Waterloo, ON, USA)^47,57^. Analyzed triplicate independent samples were compared among the treated and untreated groups of OGS. All the obtained raw data files from the mass spectrometry analysis were imported from the machine and uploaded to the PEAKS software program^47^ for quantification and interpretation of the spectra, and alignment of the total ion chromatograms along with the retention times were performed. A specific retention time of 600 to 10,500 seconds was specified. The protein identification from the raw data was performed same as we described in our earlier study^55^, an Uniprot’s reference database of Homo sapiens (release 03_2014)^58^ contained 20,272 entries were added and combined with a decoy database (the sequences were reversed) was used. The following parameters were specified for label-free quantification: digested by trypsin, with two missed cleavages; precursor mass tolerance was 10 ppm; fragment mass tolerance: 0.7 Da, minimum charge: 2, maximum charge: 3, carbamidomethylation, oxidation (M), and deamidated (N and Q) were specified as fixed and variable modifications. For determining the false-positive identification rate, the estimated spectra were used against the decoy database. The confident protein identifications and quantifications were estimated using a false discovery rate (FDR) of <1%, containing the peptide score of −10 log p ≥ 20 was employed. To determine the relative protein and peptide abundance in the tested samples, peptide feature-based quantification was performed^59^. For the accurate identification of peptide intensity differences among two samples the peptide signal intensity is directly proportional to the abundance of the peptides in the sample; hence the estimated peptide features were matched perfectly and the differences in peptide intensity between two samples were quantified effectively. Then, the area under the curve (AUC) of the extracted ion chromatograms (XICs) were measured and compared among the three analyzed runs. To determine the total cumulative peak area of the identified proteins, only unique peptides that are specifically assigned to the particular proteins were designated. FDR was calculated based on the target/decoy database as mentioned in the earlier studies^60^, and the >1% FDR peptides were chosen as true positive hits (considering the chance of getting one false positive in 20 observations). With this active feature-based quantitative approach^61^ the identified peptides with *p*-values < 0.05 and 0.01 that were identified in at least three observations from the OGS treated and untreated were compared and measured.

For identifying the significantly differential protein expressions among the tested groups’ one-way analysis of variance (ANOVA)^62^ was performed. The quantified spectral datasets were normalized with their spectral abundance factor values (triplicate experiments were averaged) that were used to generate a heat map showing the differentially expressed proteins between three groups. To minimize false positives, we excluded the proteins with an individual false detection rate *p* > 0.05 from further analysis. The quantified proteins with an XIC value lower than 100,000 were observed as absent (noise) and identified in only one of the three technical replicates were also excluded in this study. Each sample technical replicate XIC values were averaged and quantified for each OGS untreated and treated samples group, and the ratios of OGS-untreated/OGS-treated with cryotherapy, and ECIR were employed to identify the differentially expressed proteins as down-regulated proteins with <0.3–0.5 folds. Upregulated proteins were denoted with OGS-untreated/OGS-treated with a fold change of <1.5 to 2.

### Protein identification

The Mascot software program (Matrix Science version 2.2, http://www.matrixscience.com) search engine along with the UniProtKB database (UniProt release 2015-10) and National Center for Biotechnology non-redundant (NCBInr) was used for the protein identification of the analyzed samples. To distinguish the altered proteins the following parameters have been specified: trypsin was specified for the enzymatic digestion with two missed cleavages, the protein modification changes observation was employed by specifying carbamidomethyl as a constant modification, and oxidation (M) as variable modification. The peptide mass tolerance of 50 ppm, and 0.1 Da MS/MS tolerance with an FDR of <1% were used for the accurate protein observation. Based on the specified parameters the proteins that are consistently identified from all the three technical replicates or at least two of the three analyses were selected for further evaluations. The theoretical molecular mass (MW) and isoelectric point (pI) of the identified proteins from this study were determined using the Mascot database.

### Bioinformatics analysis

To understand the identified proteins involvement in various biological processes (BPs) and their molecular functions (MFs), along with the protein categories and cellular components (CCs) an international standardized gene function classification system of gene-ontology(GO) (http://www.geneontology.org/), and the DAVID (http://david.abcc.ncifcrf.gov/) (Database Annotation Visualization, and Integrated Discovery) database for functional analysis were performed^63–66^. For evaluate the protein-protein interactions among the identified proteins from OGS untreated Vs. treated we further analyzed our results using protein-protein interaction (PPI) networks with STRING (Search Tool for the Retrieval of Interacting Genes/Proteins, Version 9.1) networks (website: http://string-db.org/) and specified high score of 0.09 along with the default parameters for the significant results.

### Statistical analysis

To differentiate the changes in the protein profiles from the untreated Vs. treated OGS patients with LN-freezing/cryotherapy and ECIR, each patient sample was analyzed in a triplicate, and the variations in the percentage of volume and relative intensity were confirmed by statistical analysis. The differential expressions of the proteins quantified using spectral counting assessments for the LC-ESI-MS/MS data evaluations. Each sample was evaluated in three technical replicates and the average of the obtained abundance spectra was calculated. The data are expressed as mean ± standard deviation (SD) was determined using analysis of variance (ANOVA)^62^ assessment, and Mann-Whitney U-test was performed by SPSS statistical package (SPSS19, SPSS Ltd., Woking, Surrey, UK) for Windows. A probability value < 0.05 was considered as statistically significant and <0.01 was considered as highly significant.

### Functional annotation of protein cohort

The identified DEPs from OGS treated and untreated samples were characterized using sub-cellular localization (SC), molecular function (MF), biological process (BP) and pathway analysis were performed using GO, PANTHER version 7.1, DAVID functional enrichment, and Inequity pathway (IPA) was used^65,67^. From our analysis we have gained a better understanding and biological context of the identified proteins and their involvement with the disease and its pathobiology of involvement of various physiological pathways.

### Western blot analysis

Validation of the selected proteins was carried using western blotting analysis in another set of OGS bone tissue samples (n = 6) as described in earlier studies^68,69^. Proteins were separated by SDS-PAGE on to an electro transferred PVDF membrane (Millipore Corporation, Bedford, MA, USA) at 100 V for 60 min. In a TTBS solution [0.2 M TRIS-HCl (pH 7.6), 1.37 M NaCl, 0.1%Tween-20]^70^, the transferred protein membranes were immersed in 5% non-fat milk for 1 hr at room temperature. The proteins were incubated with primary antibodies, Fibronectin (FN) rabbit monoclonal antibody^71^ (catalog no. ab2413, 1:1000 dilution), protein S100a4 rabbit mAb (catalog ab124805, :10000 dilution), beta-actin rabbit mAb (catalog no. ab8227, 1:1000 dilution) at 4 °C overnight. All the antibodies were purchased from Abcam (www.abcam.com) (Cambridge, United Kingdom). Then the membranes were washed and incubated in 5% non-fat milk in a TTBS solution for 3 h at room temperature and subjected to three 5 min rinses in a TTBS solution. Later membranes were incubated with a horseradish peroxidase-conjugated goat anti-rabbit antibody (Zhongshan Golden Bridge Biotechnology Co., Ltd, Beijing, China; catalog no. 7074) for 1 h at room temperature, and subjected to three 5 min rinses in a TTBS solution. The blot was developed with a Super ECL Plus kit (Applygen, Beijing, China), and the signal was exposed with X-ray film. The images were scanned, and the intensity of each band was captured using an Image Master 2D Platinum version 5.0 (GE Healthcare Amersham Bioscience). The intensity of each band was standardized as a percentage of the total intensity and the results were referred to as a relative volume that represents the relative expression abundance of the gene in the samples tested. The relative expression abundance was used to evaluate protein expression stability. Western blotting and quantification analysis were performed in at least three biological replications.

### Ethics approval

This study was approved from the institutional review board of VGH-TPE, Taiwan (**IRB Approval no.2019-02-021 A**). And an informed consent was obtained from all the patients.

## Supplementary information

Dataset 1

Dataset 2

## Data Availability

All the triplicated datasets generated during and/or analyzed during the current study are available from the corresponding author upon request. However, the required data which was analyzed in this study are included in Supplementary Information Files.
